# Evaluating Chlorine Sanitization at Practical Concentrations for Controlling *Listeria monocytogenes* and *Salmonella* on Fresh Peaches

**DOI:** 10.3390/foods13213344

**Published:** 2024-10-22

**Authors:** Xiaoye Shen, Mengqian Hang, Yuan Su, Jeanene Marie de Avila, Mei-Jun Zhu

**Affiliations:** School of Food Science, Washington State University, Pullman, WA 99164, USA; xiaoye.shen@wsu.edu (X.S.); mengqian.hang@wsu.edu (M.H.); yuan.su@wsu.edu (Y.S.); deavilaj@wsu.edu (J.M.d.A.)

**Keywords:** peach, chlorine, sanitization, *Listeria monocytogenes*, *Salmonella*, cross-contamination

## Abstract

Recent foodborne outbreaks and recalls involving *Listeria monocytogenes* and *Salmonella*-contaminated peaches have caused significant economic losses to the peach industry. This study evaluated the effectiveness of chlorine, a commonly used sanitizer in the fresh produce industry, against *L. monocytogenes* and *Salmonella* and its ability to control cross-contamination in fresh peaches. Peaches inoculated with *L. monocytogenes* or *Salmonella* (~6 log_10_ CFU/peach) were treated with 50–150 mg/L of free chlorine (FC, pH6.8) 24 h post-inoculation. The results revealed that chlorine had similar efficacy against *L. monocytogenes* and *Salmonella* on peaches (*p* > 0.05). A 30 s treatment at 50, 100, and 150 mg/L FC resulted in dose-dependent reductions (*p* < 0.05), achieving reductions of 0.88–0.92, 1.54–1.61, and 1.73–1.79 log_10_ CFU/peach, respectively. Extending the contact time to 2 min slightly but significantly enhanced the chlorine efficacy (*p* < 0.05). Additionally, a 30 s to 2 min exposure to chlorine with 50–150 mg/L FC resulted in a 1.05–1.43 log_10_ CFU/peach reduction in yeasts and molds. Tap water exposure led to substantial cross-contamination between inoculated and uninoculated fruits and processed water, with *Salmonella* exhibiting higher transfer rates than *L. monocytogenes*. The application of chlorine mitigated the cross-contamination of both pathogens but did not entirely prevent it. These findings offer valuable insights for the peach and other stone fruit industries to verify process controls.

## 1. Introduction

The United States ranks as the fourth-largest global producer of peaches, with 669,000 metric tons in 2024, behind China, the EU, and Turkey [1]. *Listeria monocytogenes* is a major foodborne pathogen that presents a severe health risk, particularly to vulnerable groups. While infection linked to *L. monocytogenes* in the tree fruits are rare, two *Listeria* outbreaks have linked to stone fruits [2,3,4]. A recent *L. monocytogenes* outbreak connected to stone fruits resulted in 11 illnesses and one death from 2018 to 2023 [2]. Additionally, in 2020, an outbreak was linked to fresh peaches due to the potential *Salmonella* Enteritidis contaminations [5,6,7]. These outbreaks highlight the need of the effective intervention strategies to ensure the microbial safety of peaches.

Peaches are susceptible to contamination by foodborne pathogens at various stages of handling. Before reaching packinghouses, contamination by foodborne pathogens present in the natural environment may occur, such as those in soil, water, and wildlife [8,9]. Cross-contamination is also a concern within packinghouses. *Listeria* spp., which are ubiquitous in environment, was detected on a packer’s hand and the cold room floor in a peach packinghouse [10]. 

To mitigate the risk of cross-contamination, antimicrobial sanitizer interventions are commonly applied during the post-harvest packing of peaches within packinghouses. Chlorine is the predominant choice in fresh produce packing [11], particularly in the stone fruit packing line (personal communication with the stone fruit industry), likely owing to its cost-effectiveness and no effect on fruit quality. The current recommended practice for chlorine in fruit packinghouses involves maintaining a constant concentration of 100–150 mg/L free chlorine (FC), with a pH range of 6.5–7.5 in fruit wash water [12]. However, its efficacy in controlling foodborne pathogens varies based on the type of produce and the specific pathogen targeted. For example, a 30 s chlorine intervention with 70 mg/L FC led to reductions in *Salmonella* and *L. monocytogenes* on cantaloupes by 2.5 and 1.4 log_10_ CFU/cantaloupe, respectively [13]. Conversely, on cherry tomatoes, chlorine showed higher efficacy against *L. monocytogenes* than *Salmonella*. A 3 min treatment with 200 mg/L FC resulted in a 1.78 log_10_ CFU/g reduction in *Salmonella* and a 4.10 log_10_ CFU/g reduction in *L. monocytogenes* on cherry tomatoes [14]. Additionally, its effectiveness is influenced by the level of organic matter present in the water [15] and water hardness [16,17]. However, little is known about the efficacy of chlorine intervention against *Salmonella* and *L. monocytogenes* on fresh peaches or other stone fruits. Only one study reported that a 5 s chlorine wash at 22 mg/L (pH 8.2) caused a 1.4 log_10_ CFU/peach reduction in *Listeria innocua* on Sweet September peaches [18].

This study, therefore, aimed to investigate the efficacy of chlorine intervention within the concentration ranges commonly applied in the stone fruit packing line against *L. monocytogenes* and *Salmonella* for pathogen reduction and the prevention of cross-contamination, providing practical and actionable information for the stone fruit industry.

## 2. Materials and Methods

### 2.1. Bacterial Strains and Inoculum Preparation

The *L. monocytogenes* NRRL B-33053 (1983 coleslaw outbreak isolate), NRRL B-33466 (processing plant isolate), and NRRL B-57618 (2011 cantaloupe outbreak isolate) were obtained from the USDA-ARS culture collection (National Center for Agricultural Utilization Research (NRRL), Peoria, IL, USA). *Salmonella* Enteritidis PT30 was obtained from Dr. Linda Harris (University of California, Davis), and *Salmonella* Tennessee K4643 and *Salmonella* Agona 447967 were obtained from Dr. Nathan Anderson (USDA, Greater Chicago, IL, USA). All stains were maintained at −80 °C in trypticase soy broth (Becton, Dickinson and Company (BD), Sparks, MD, USA) supplemented with 0.6% yeast extract (TSBYE) and 20% glycerol.

Each frozen stock culture was resuscitated in TSBYE at 37 °C twice, washed in sterile phosphate-buffered saline (PBS, pH 7.4), and combined in equal volumes to obtain a 3-strain cocktail of *L. monocytogenes* or *Salmonella* for peach inoculation.

### 2.2. Peach Inoculation

Yellow-flesh peaches, harvested at commercial maturity, were obtained from a commercial peach packing facility in Fresno, CA. Peaches, without cuts or bruises, were washed with tap water, gently wiped with tissue paper to remove surface fuzz, and dried at room temperature (~22 °C, RT) before inoculation. The peaches were then inoculated with the 3-strain *L. monocytogenes* or *Salmonella* cocktail at ~10^6^ CFU/peach, and underwent antimicrobial treatments 24 h after inoculation. Ten peaches were sampled right after inoculation and 24 h post-inoculation to determine the population of *L. monocytogenes* or *Salmonella* on the peaches.

### 2.3. Preparation of Chlorine Solution

The chlorine was prepared from Accu-Tab (Pace International, Wapato, WA, USA) in tap water to achieve FC concentrations of 50, 100, and 150 mg/L. The hardness of the water used in this study was 120 ± 0 ppm, as determined using a total hardness test kit (Hach, Loveland, CO, USA). The FC level was below the limit of detection (LOD, 0.2 ppm), as measured with the Taylor K-2006 kit (Taylor Technologies, Sparks, MD, USA). The pH of the chlorine solutions was adjusted to 6.8 using 6 mol/L HCl and verified with an Orion Ultra pH/ATC Triode (Thermo Fisher Scientific, Waltham, WA, USA). The level of FC was confirmed using the Taylor K-2006 test kit (Taylor Technologies, Sparks, MD, USA) immediately after the preparation of each sanitizer solution.

### 2.4. Chlorine Intervention on Peach Surfaces

A set of ten inoculated peaches was submerged in respective chlorine intervention for 30 s or 2 min at RT. The tap water wash was used as a control. Additionally, a set of uninoculated peaches underwent chlorine submersion intervention at the same concentration for the evaluation of yeasts and molds. Following the intervention, the peaches were transferred to sterile stomach bags (Thermo Fisher Scientific) to determine the viable bacterial populations. Each experiment was independently repeated three times.

### 2.5. Evaluation of Cross-Contamination

To further assess the efficacy of chlorine in controlling the cross-contamination of peaches, the inoculated peaches were introduced alongside uninoculated peaches at a ratio of 1:4 into chlorine solutions with 50–150 mg/L of FC for 30 s or 2 min at RT. Immediately after the intervention, each inoculated or uninoculated peach was individually transferred to a sterile stomach bag for the determination of viable bacterial populations. The bacterial counts in the spent wash water were also determined. Each experiment was independently repeated three times.

### 2.6. Bacterial Detachment and Enumeration

To enumerate the bacterial populations on inoculated and uninoculated peaches, 10 mL of neutralization buffer (BD) was added to each stomacher bag containing a peach, which was then hand rubbed for 120 s. The detached bacterial suspension was serially diluted and plated onto duplicate TSAYE (TSBYE with 1.5% agar) plates overlaid with Modified Oxford Agar (MOX, BD) for *L. monocytogenes* and Xylose Lysine Deoxycholate Agar (XLD, BD) for *Salmonella*. The plates were then incubated at 37 °C for 48 h.

The residual bacterial populations in spent wash solutions were enumerated following the above-mentioned quantitative method. Additionally, the membrane filtration method was used for determining the residual bacterial populations in spent wash solutions, where 100 mL of spent chlorine solution was filtrated through a 0.2 μm analytical test filter funnel (Thermo Fisher Scientific) and followed by a rinse with neutralizing buffer. The membrane was then placed on TSAYE plates overlaid with MOX for *L. monocytogenes* and XLD for *Salmonella*, and incubated at 37 °C for 48 h. For enrichment, 1 mL of neutralized spent wash solution was transferred into 9 mL of buffered *Listeria* enrichment broth (BD) and Rappaport Vassiliadis *Salmonella* enrichment broth (Thermo Fisher Scientific) for *L. monocytogenes* and *Salmonella*, respectively. The enrichments were then incubated at 30 °C for 48 h for *L. monocytogenes* and 42 °C for 48 h for *Salmonella*.

### 2.7. Yeasts and Molds Enumeration

The rub solutions of uninoculated peaches after their respective chlorine intervention were serially diluted and the appropriate dilution was plated onto potato dextrose agar (PDA, BD) plates to determine the yeast and mold counts. The PDA plates were incubated at RT for 5 days.

### 2.8. Statistical Analysis

The data were reported as means ± standard error mean (SEM) averaged from three independent experiments with 10 peaches per treatment or two wash water samples per treatment in each independent study, unless specified. Statistical analysis was conducted using one-way analysis of variance (ANOVA) followed by Tukey’s multiple comparisons using IBM SPSS version 20 (Chicago, IL, USA). *p* values equal to or less than 0.05 were considered statistically significant.

## 3. Results

### 3.1. Efficacy of Chlorine Intervention against L. monocytogenes

**Figure 1:**
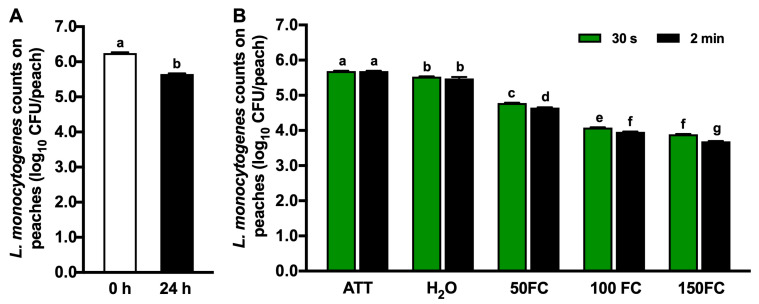
Efficacy of chlorine against *Listeria monocytogenes* on peaches with 30 s or 2 min contact. (**A**) *L. monocytogenes* counts on peaches immediately after inoculation (0 h) and 24 h post-inoculation (24 h). (**B**) *L. monocytogenes* recovered on peaches after 30 s or 2 min contact in the respective wash solution. ATT: peaches 24 h post-inoculation. H_2_O: peaches washed with tap water. FC: free chlorine. 50 FC, 100 FC, or 150 FC: peaches washed with chlorine solution at 50 mg/L FC, 100 mg/L FC, or 150 mg/L FC. Mean ± SEM averaged from three independent studies with 10 peaches/treatment in each independent study. Histogram bars without common letters differ significantly (*p* < 0.05).

The initial inoculation level of *L. monocytogenes* on peaches was 6.25 log_10_ CFU/peach (Figure 1A). Following a 24 h attachment at RT, the count of *L. monocytogenes* decreased to 5.65 log_10_ CFU/peach (*p* < 0.05) (Figure 1A). The tap water wash resulted in 0.15–0.24 log_10_ CFU/peach reductions after 30 s or 2 min of contact (Figure 1B). A 30 s exposure to chlorine at 50 mg/L FC resulted in a reduction in *L. monocytogenes* by 0.92 log_10_ CFU/peach (Figure 1B). The efficacy of chlorine improved with an increasing concentration (*p* < 0.05); FC at 100 and 150 mg/L caused 1.61 and 1.79 log_10_ CFU/peach reductions in *L. monocytogenes* after 30 s (Figure 1B). Extending the contact time from 30 s to 2 min resulted in a slight but significant improvement in the antimicrobial efficacy of chlorine against *L. monocytogenes* at their respective concentrations (Figure 1B, *p* < 0.05).

### 3.2. Efficacy of Chlorine Intervention against Salmonella

**Figure 2:**
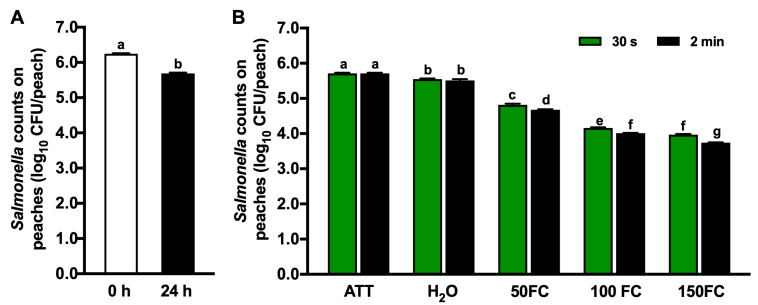
Efficacy of chlorine against *Salmonella* on peaches with 30 s or 2 min contact. (**A**) *Salmonella* counts on peaches immediately after inoculation (0 h) and 24 h post-inoculation (24 h). (**B**) *Salmonella* counts recovered on peaches after 30 s or 2 min contact. ATT: peaches 24 h post-inoculation. H_2_O: peaches washed with tap water. FC: free chlorine. 50 FC, 100 FC, or 150 FC: peaches washed with chlorine solution at 50 mg/L FC, 100 mg/L FC, or 150 mg/L FC. Mean ± SEM averaged from three independent studies with 10 peaches/treatment in each independent study. Histogram bars without common letters differ significantly (*p* < 0.05).

The inoculation level of *Salmonella* on peaches and the counts 24 h after inoculation were comparable to those of *L. monocytogenes* (Figure 2A). Likewise, tap water wash had a limited efficacy in reducing *Salmonella* on peaches, regardless of the contact time (Figure 2B). Chlorine led to a dose-dependent reduction in *Salmonella* on peaches (*p* < 0.05) (Figure 2B). After 30 s of contact with 50, 100 and 150 mg/L FC, the viable count of *Salmonella* on peaches reduced by 0.88, 1.54 and 1.73 log_10_ CFU/peach, respectively (Figure 2B). Noticeably, the magnitude of pathogen reduction became smaller after the FC reached 100 mg/L, irrespective of the pathogen type. Increasing the contact time from 30 s to 2 min resulted in an additional reduction in *Salmonella* on peaches by 0.15, 0.15, and 0.23 log_10_ CFU/peach for the chlorine intervention with 50, 100, and 150 mg/L of FC, respectively (*p* < 0.05, Figure 2B). Additionally, chlorine at the tested concentrations showed comparable efficacy against *L. monocytogenes* and *Salmonella* at each concentration (*p* > 0.05, Figure 1B and Figure 2B).

### 3.3. Effectiveness of Chlorine Intervention in the Prevention of Cross-Contamination

During the 30 s to 2 min exposure in tap water, *L. monocytogenes* transferred from inoculated peaches to uninoculated peaches and water by 2.87–2.91 log_10_ CFU/peach and 1.73–1.91 log_10_ CFU/mL, respectively (Table 1). A higher rate of *Salmonella* transfer from inoculated fruits to uninoculated fruits (3.01 log_10_ CFU/peach) and wash water (2.31–2.41 log_10_ CFU/mL) was observed in tap water without sanitizers (Table 2). The transfer of bacteria to uninoculated fruit and water decreased with the increased chlorine concentration, regardless of the contact time (Table 1 and Table 2). *L. monocytogenes* was transferred from inoculated peaches to uninoculated peaches by 2.65–2.67, 2.45–2.52, and 2.00–2.14 log_10_ CFU/peach in chlorinated water with 50, 100, and 150 mg/L FC, respectively, after up to 2 min of contact time. The levels recovered in the spent wash water were 0.68–0.73 log_10_ CFU/mL, 1.00–1.17 log_10_ CFU/100 mL, and ~1 log_10_ CFU/100 mL, respectively (Table 1). The efficacy of chlorine in controlling the cross-contamination of *Salmonella* from inoculated fruits to uninoculated fruits was not different from that of *L. monocytogenes* (*p* > 0.05) (Table 1 and Table 2). However, the counts of *Salmonella* recovered in the spent wash solution were lower than those of *L. monocytogenes* (Table 1 and Table 2).

### 3.4. Efficacy of Chlorine Intervention against Yeasts and Molds on Peaches

**Figure 3:**
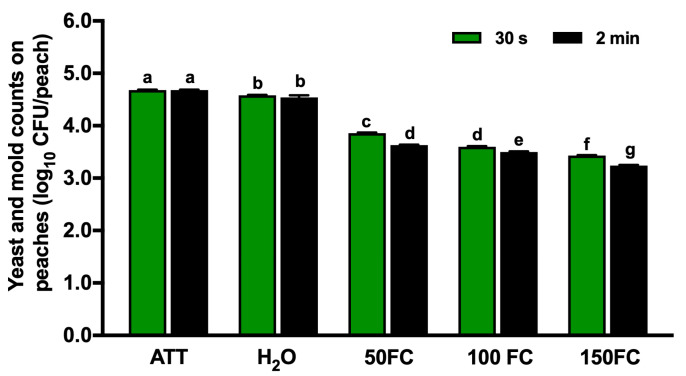
Efficacy of chlorine against yeasts and molds on uninoculated peaches after 30 s or 2 min contact. H_2_O: peaches washed with tap water. FC: free chlorine. 50 FC, 100 FC, or 150 FC: peaches washed with chlorine solution at 50 mg/L FC, 100 mg/L FC, or 150 mg/L FC. Mean ± SEM averaged from three independent studies with 10 peaches/treatment in each independent study. Histogram bars without common letters differ significantly (*p* < 0.05).

The native yeast and mold count on peaches was 4.68 log_10_ CFU/peach. A tap water wash of up to 2 min had a limited ability to remove yeasts and molds, achieving only a ~0.1 log_10_ CFU/peach reduction (Figure 3). The application of chlorine and the extension of the contact time at the respective chlorine concentrations improved (*p* < 0.05) the removal of yeasts and molds. Exposure to 50, 100, and 150 mg/L of FC for 30 s to 2 min reduced yeasts and molds by 0.81–1.05, 1.07–1.17, and 1.24–1.43 log_10_ CFU/peach, respectively (Figure 3).

## 4. Discussion

### 4.1. Attachment of Salmonella and L. monocytogenes on Fresh Peaches

Following a 24 h attachment at RT after inoculation, the *L. monocytogenes* levels on peaches were reduced by 0.5 log_10_ CFU/peach from an initial level of 6.25 log_10_ CFU/peach. This attachment pattern was mirrored by *Salmonella* on peaches. Similar to our findings, Yan et al. [19] reported a reduction in *E. coli* O157:H7 by ~1.0 log_10_ CFU/fruit on fuzz-removed peaches, following an initial inoculation of ~8.5 log_10_ CFU/fruit 24 h after inoculation [19]. In contrast, the populations of *L. monocytogenes* on peaches with natural fuzz, initially inoculated at ~5.7 log_10_ CFU/fruit, remained unchanged after a 20 h holding period at 18–20 °C [20]. In a similar vein, *L. monocytogenes* exhibited no change, while *Salmonella* increased on peaches with natural fuzz after a 24 h holding period at 21 °C [21]. The exact reason for the distinct survival and attachment outcomes of *L. monocytogenes* and *Salmonella* on peaches remains unknown. This variability could be attributed to factors such as differences in strains, inoculation methods, the surrounding environment, and their complex interactions. Additionally, peach surfaces are naturally covered by trichomes, known as fuzz. The removal of these trichomes may contribute to the observed difference, as it can leave scars where they are uprooted, potentially creating hidden inches for bacteria.

### 4.2. Efficacy of Chlorine against Foodborne Pathogens on Fresh Peaches

The chlorine treatment showed a distinct dose-dependent response against both *L. monocytogenes* and *Salmonella* on peaches. This aligns with the findings that treatment with 25–100 mg/L FC is concentration-dependent in controlling *L. monocytogenes* on apples [22]. Likewise, *Salmonella* was reduced by 0.6, 1.2, and 1.3 log_10_ CFU/cm^2^ on green tomatoes following a 2 min chlorine intervention with 60, 110, and 210 mg/L FC, respectively [23]. However, the reduction in *L. monocytogenes* on peaches (>1.5 log/peach with 100 ppm FC) was higher than on apples (~1 log/apple with 100 ppm FC) [22,24]. This difference could be due to variations in the produce type, the attachment time between inoculation (24 h vs 48 h) [22,24], and the sanitizer interventions. The antimicrobial mechanism of chlorine involves disrupting the cell membrane, denaturing intracellular enzymes and other important macromolecules, and interfering with the critical biosynthesis pathways in bacterial cells [25].

The antimicrobial effect of chlorine increased with an increase in the exposure time for both *L. monocytogenes* and *Salmonella*. However, the extent of pathogen reduction varies depending on factors such as the produce type, concentration, contact time, inoculation method, and others. For instance, increasing the exposure time from 5 s to 30 s caused a 2-log higher reduction in *Salmonella* on tomatoes treated with 100 mg/L FC, where *Salmonella* was spot-inoculated on tomatoes with a 2 h attachment at RT [26]. Increasing the exposure time from 5 s to 40 min for peaches spot-inoculated with *L. innocua*, held overnight at 4 °C, and treated with 21 mg/L FC resulted in additional 1.6 log_10_ CFU/peach reductions [18]. Exposure to chlorine for 50 s and 90 s with 50–150 mg/L FC led to 2.3–2.5 and 2.5–2.8 log_10_ CFU/g log reductions in *Salmonella* on lettuce, respectively [27]. Similarly, the populations of *L. monocytogenes* were reduced by 2.0 and 3.0 log_10_ CFU/strawberry on strawberries exposed to 200 mg/L FC for 60 s and 120 s, respectively [28].

In our study, chlorine at the tested concentrations exhibited a comparable antimicrobial efficacy against *L. monocytogenes* and *Salmonella* on fresh peaches, consistent with the findings for tomatoes treated with 200 mg/L FC [29]. However, Lacombe and Wu [30] found a 1.5 log higher reduction in *Listeria* compared to *Salmonella* on blueberries treated with 200 mg/L FC. While *Salmonella*, a Gram-negative bacterium, possesses an outer membrane, potentially leading to higher resistance to chlorine, the complex nature of pathogen attachment to produce surfaces contributes to varying sanitation outcomes on different produce.

On the other hand, a tap water wash showed limited effectiveness in reducing microbial contamination, with a 2 min wash reducing both pathogens by 0.1–0.2 log_10_ CFU/peach. This aligns with previous findings, where a 2 min tap water wash reduced the *L. monocytogenes* on apples by a similar amount [24,31], and a 1 min water wash reduced the *L. innocua* on lettuce by ~0.2 log_10_ CFU/g [32]. Water wash processing can release loosely attached pathogens from produce into process water, potentially serving as a source of cross-contamination. This underscores the importance of implementing proper sanitization measures and water management strategies in postharvest produce processing and packing to minimize the risk of cross-contamination.

### 4.3. Effect of Chlorine in Preventing Cross-Contamination

In the absence of chlorine during peach washing, ~3 log of *L. monocytogenes* and *Salmonella* spread from inoculated peaches (initial level of ~6 log) to uninoculated peaches. Similarly, during a 2 min exposure in water, the transfer of 3.6–4.2 log of *L. monocytogenes* from inoculated apples to uninoculated apples occurred, depending on the inoculated-to-uninoculated fruit ratio and the condition of the process water [22,24]. The application of chlorine reduced the transfer of bacteria from inoculated peaches to uninoculated peaches and into wash water in a concentration-dependent manner. These results mirrored findings in studies on fresh apples, where the effectiveness of preventing *L. monocytogenes* cross-contamination between inoculated apples, uninoculated apples, and process water increased with a higher FC concentration (25 to 100 mg/L) [22] or JC 9450, a chlorine-based sanitizer (0.125% to 0.50%) [24]. Similarly, the incidence of *Salmonella* cross-contamination on winter jujube decreased as the initial chlorine concentration increased from 5 to 10 mg/L FC [33]. Maintaining an effective FC level in wash water during produce processing is critical for controlling cross-contamination. Additionally, the initial contamination level of fruits, the produce-to-water ratio, the water replenishment rate, and the contact time are key factors influencing the efficacy of chlorine during fruit processing [34,35,36].

Despite comparable populations of *L. monocytogenes* and *Salmonella* being detected on uninoculated peaches after up to 2 min of contact, higher counts of *Salmonella* were recovered from the spent wash water compared to *L. monocytogenes* during the tap water-only wash. While the exact reason for this remains unclear, Cabrera-Diaz et al. [37] reported that *L. monocytogenes* demonstrated a slightly higher attachment strength to fruit than *Salmonella*, which could partially explain the lower recovery of *L. monocytogenes* in wash water. Interestingly, while higher levels of *Salmonella* were detected in water during the water-only wash compared to *L. monocytogenes*, lower counts of *Salmonella* were recovered in chlorinated water, regardless of concentration, in comparison to *L. monocytogenes*. The exact mechanism for this is unknown but may be related to differences in the structural and attachment capabilities of the pathogens on peaches. In alignment with our findings, Krishnan et al. [38] reported that *L. monocytogenes* require a longer duration than *Salmonella* to achieve the same reduction in surface water when treated with chlorine at the same concentration. The intricate nature of chlorine-mediated cross-contamination prevention warrants further examination under various scenarios or in industrial conditions. To ensure measurable pathogen reduction, a high pathogen inoculation level was used in this study. While this is a standard approach used in controlled laboratory settings to evaluate the efficacy of sanitizers, it is higher than the natural contamination loads typically found in real-world conditions, as reported by Chen et al. [4]. Future studies should consider testing with lower inoculation levels to better reflect commercial conditions.

### 4.4. Efficacy of Chlorine against Yeasts and Molds on Peaches

The native yeast and mold levels on the peaches utilized in this study were comparable to those reported by Sy et al. [39] at ~4.8 log_10_ CFU/peach, while surpassing the counts reported by Wang et al. [40] at 2.3 log_10_ CFU/g from a Georgia packinghouse. The factors contributing to these differences may include varietal distinctions, geographical locations, preharvest practices, and postharvest handlings. Notably, the peaches in our study underwent overnight ambient temperature transportation, which might partially explain the relatively high levels of yeasts and molds.

The yeast and mold counts were reduced by 1.1–1.4 log_10_ CFU/peach after a 2 min wash in chlorinated water with 50–150 mg/L FC. Likewise, a hydrocool process involving water supplemented with 150–200 mg/L FC at pH 7.0–7.3 in a shower hydrocooler maintained at 2–5 °C resulted in a ~2.1 log_10_ CFU/peach reduction in yeasts and molds after one hour of contact [41]. Studies on fresh apples revealed that 2 min of contact with a ~100 mg/L FC solution resulted in a ~0.6 log_10_ CFU/apple reduction in yeasts and molds on Granny Smith apples [24]. A 5 min exposure to 50 and 100 mg/L chlorine wash interventions led to reductions in yeasts and molds by 0.7 and 1.1 log_10_ CFU/g on fresh tomatoes [42]. However, Wang et al. [43] found that a chlorinated water spray wash with 50 mg/L of FC for 20 s, followed by waxing, did not impact the yeast and mold counts on peaches. These findings underscore the complexity of sanitation methods and the need for tailored approaches based on specific produce types and treatment conditions.

## 5. Conclusions

Chlorine application showed similar efficacy against *L. monocytogenes* and *Salmonella* on fresh peaches. Chlorine at 50, 100, and 150 mg/L FC resulted in reductions in *L. monocytogenes* and *Salmonella* by ~0.9, 1.5–1.6, and 1.7–1.8 log_10_ CFU/peach, respectively, after 30 s. Extending the contact time from 30 s to 2 min led to a slight but significant increase in efficacy against both pathogens. A 2 min exposure to 50–150 mg/L of FC caused 1.1–1.4 log_10_ CFU/peach reductions in yeasts and molds. The tap water wash resulted in substantial cross-contamination, particularly with *Salmonella*, while the chlorine interventions significantly reduced but did not completely prevent bacterial transfer. It is worth pointing out that the contamination levels in this study were intentionally higher than the practical contamination levels to obtain log reductions. These findings provide useful information for the stone fruit industry to enhance food safety during postharvest packing.

## Figures and Tables

**Table 1:** Efficacy of chlorine intervention in preventing the cross-contamination of *Listeria monocytogenes* from inoculated peaches to uninoculated peaches and washing solutions.

Contact Time	Treatment	Inoculated Fruit	Uninoculated Fruit	Recovered in Spent Wash Solution	
		Reduction (log_10_ CFU/Peach)	Recovery (log_10_ CFU/Peach)	Plating (log_10_ CFU/mL)	MF (log_10_ CFU/100 mL)
30-s	Tap water	0.12 ± 0.03 ^a^	2.87 ± 0.01 ^a^	1.73 ± 0.04 ^a^	/
	50 mg/L FC	0.94 ± 0.02 ^b^	2.65 ± 0.06 ^b^	0.73 ± 0.09 ^b^	/
	100 mg/L FC	1.68 ± 0.01 ^d^	2.52 ± 0.01 ^c^	/	1.17 ± 0.04 ^a^
	150 mg/L FC	1.83 ± 0.02 ^e^	2.14 ± 0.11 ^d^	/	0.98 ± 0.09 ^a^
2-min	Tap water	0.17 ± 0.04 ^a^	2.91 ± 0.01 ^a^	1.91 ± 0.01 ^c^	/
	50 mg/L FC	1.06 ± 0.02 ^c^	2.67 ± 0.04 ^b^	0.68 ± 0.10 ^b^	/
	100 mg/L FC	1.76 ± 0.02 ^e^	2.45 ± 0.06 ^c^	/	1.00 ± 0.08 ^a^
	150 mg/L FC	2.00 ± 0.02 ^f^	2.00 ± 0.12 ^d^	/	0.99 ± 0.07 ^a^

MF: membrane filtration. FC: free chlorine. Each intervention was independently repeated three times. Means ± SEM, n = 9 for inoculated peaches, n = 36 for uninoculated peaches, and n = 6 for wash water samples. ^a–f^ means within a column with different letters differ significantly (*p* < 0.05).

**Table 2:** Efficacy of chlorine intervention in preventing the cross-contamination of *Salmonella* from inoculated peaches to uninoculated peaches and washing solutions.

Contact Time	Treatment	Inoculated Fruit	Uninoculated Fruit	Recovered in Spent Wash Solution	
		Reduction (log_10_ CFU/Peach)	Recovery (log_10_ CFU/Peach)	Plating (log_10_ CFU/mL)	MF (log_10_ CFU/100 mL)
30-s	Tap water	0.15 ± 0.02 ^a^	3.01 ± 0.06 ^a^	2.41 ± 0.02 ^a^	/
	50 mg/L FC	0.99 ± 0.02 ^b^	2.72 ± 0.04 ^b^	/	0.57 ± 0.17 ^a^
	100 mg/L FC	1.67 ± 0.01 ^d^	2.67 ± 0.06 ^bc^	/	0.18 ± 0.11 ^b^
	150 mg/L FC	1.86 ± 0.01 ^f^	2.56 ± 0.08 ^bc^	/	0.00 ± 0.00 ^b^
2-min	Tap water	0.21 ± 0.04 ^a^	3.01 ± 0.04 ^a^	2.31 ± 0.13 ^a^	/
	50 mg/L FC	1.11 ± 0.01 ^c^	2.71 ± 0.09 ^bc^	/	0.15 ± 0.07 ^b^
	100 mg/L FC	1.79 ± 0.01 ^e^	2.56 ± 0.03 ^c^	/	0.26 ± 0.18 ^b^
	150 mg/L FC	2.03 ± 0.02 ^g^	2.38 ± 0.08 ^d^	/	0.23 ± 0.13 ^b^

MF: membrane filtration. FC: free chlorine. Each intervention was independently repeated three times. Means ± SEM, n = 9 for inoculated peaches, n = 36 for uninoculated peaches, and n = 6 for wash water samples. ^a–g^ means within a column with different letters differ significantly (*p* < 0.05).

## Data Availability

The original contributions presented in the study are included in the article, further inquiries can be directed to the corresponding author.
